# A Central Hospital Board for London

**Published:** 1920-05-15

**Authors:** 


### The Hospital

The Workers' Newspaper of Administrative Medicine and Institutional Life, Administration, National Insurance and Health.

No. 1771, Vol. LXVIII. SATURDAY, MAY 15, 1920. PRICE TWOPENCE

### A CENTRAL HOSPITAL BOARD FOR LONDON.

It has been announced by the Ministry of Health that it is intended to provide new hospitals throughout the country, but apparently no decision has yet been come to by the Ministry as to what is to be the position of the existing voluntary hospitals. The great value of their work is recognised on all hands, so fully recognised that it is taken for granted and the daily press has paid in the past comparatively little attention to hospital work. From time to time an account is given of the annual meetings of some of the great Funds, the speeches of prominent men are reported, but the account of any hospital meeting, though the work carried on in the institution is undoubtedly work of vast importance to the general health of the community, is generally summed up in a very short paragraph.

There are evidences of considerable change, and the leading newspapers of the daily press have recently directed public attention to the problem of hospitals. An important communication appeared in *The Times* of May 7 over the signature of a gentleman who has given a great deal of consideration to these matters. In his letter Mr. Asher calls attention to the imperative necessity of some kind of uniformity of action on the part of the hospitals and suggests that this could be secured by setting up a General Hospitals' Board for London. The question of the establishment of a Board has been engaging the careful attention of the leading hospital managers in London for some considerable time past. The British Hospitals Association meets from time to time and discusses various problems of hospital management, but the association includes representatives of all parts of Great Britain. Country hospitals differ materially from the London hospitals; the London hospitals again differ very considerably from one another, insomuch as some are general hospitals with medical schools, others have no medical schools and still another class are special hospitals only. Every hospital in want of funds appeals to the public. A supporter may be perfectly willing to make a donation to a hospital and have the immediate intention of doing so. He reads an appeal asking for funds and feels that it is deserving. Before he has sent his cheque yet another appeal meets his eye, still more pathetically worded, and he feels that it would be a pity to deprive the second hospital of what he primarily intended to give to the first hospital. Before he has made his donation yet another appeal strikes him, and so on. One cannot but sympathise with the dilemma in which such a donor is placed.

Do not let us forget that everyone of these hospitals is spending large sums of money on making known its needs. Each is appealing to practically the same public. If the Hospital Board for London which we understand has already been organised by the British Hospitals Association can draw up a scheme which will check and replace this cut-throat policy of extravagance much money will be saved and we should be on the high road to efficiency. Central collecting is already efficiently carried out by (1) King Edward's Hospital Fund, appealing to the city and the more wealthy donors to hospitals; (2) The League of Mercy as an offspring of this fund, appealing to persons of more moderate means; (3) The Hospital Sunday Fund, appealing through the churches; (4) The Hospital Saturday Fund, appealing to the workmen and organising workshop collections. If this Central Board for London has the initiative and the strength to secure the co-operation of all the hospitals in giving assistance to the utmost of their power to these Central Funds all those supporters of hospitals who can only be influenced by organised canvass should be reached by this means.

It is often pointed out that a provincial hospital secures the support of the town in which it stands, and of the surrounding country, comparatively easily because it represents all the hospital needs of that town and donors definitely understand that they must give to that hospital to ensure that medical and surgical aid should be available for those who may need it in the district in which they live. In

London there is no such corporate existence. No one hospital has a prescriptive right to appeal to inhabitants outside its own district. The larger hospitals are naturally placed where they can be of the greatest service to those who need to resort there for medical and surgical treatment and they have not the wealthy clientele in their district to whom they can confidently turn for assistance. If the public can be educated to realise that they owe to the hospital the efficiency of the training, both of medical men and nurses, on which they must themselves depend in time of sickness, they should the more readily give to the general funds to be equitably distributed amongst the hospitals. This scheme would not prevent each hospital making a direct appeal for assistance from those who come into personal contact with it, but it would prevent the heavy expenditure which is so often incurred in promoting a special hospital appeal, and such money as is now spent on appeals would be available for its proper purpose, the treatment of the poor and the investigation of disease.

The enormous advantages of promoting a unity of policy in the hospitals would be another result of the establishment of a strong Central Board, but we do not propose to deal with this point at the present time, believing that the question of finance is so all important as to secure for this Central Board the support of all who are really interested in the voluntary hospital principle.

